# Demonstration of an optical dentin hardness measuring device using bovine dentin with different demineralization times

**DOI:** 10.1117/1.JBO.27.10.105004

**Published:** 2022-10-22

**Authors:** Sota Kondo, Hisanao Hazama, Yutaka Tomioka, Atsushi Mine, Satoshi Yamaguchi, Saeko Okumura, Hiroaki Tanimoto, Kenzo Yasuo, Kazushi Yoshikawa, Kazuyo Yamamoto, Kunio Awazu

**Affiliations:** a Osaka University, Graduate School of Engineering, Osaka, Japan; b Osaka University, Graduate School of Dentistry, Department of Fixed Prosthodontics, Osaka, Japan; c Osaka University, Graduate School of Dentistry, Department of Biomaterials Science, Osaka, Japan; d Osaka Dental University, Department of Operative Dentistry, Osaka, Japan; e Osaka University, Global Center for Advanced Medical Engineering and Informatics, Osaka, Japan

**Keywords:** dentin, hardness, light-emitting diode, dark area, root caries, demineralization

## Abstract

**Significance:**

The increase in root caries is a serious problem as society ages. Root caries is diagnosed by inspection and palpation, which are qualitative. A method to objectively and quantitatively evaluate the progress of root caries in a clinical setting is strongly desired. The root caries could be diagnosed by measuring hardness because dentin becomes softer as the caries progresses. Vickers hardness has been customarily used as an indicator of tooth hardness. However, this method cannot be used to *in vivo* teeth because the teeth must be dried prior to measurement to make the indentation. A hardness meter using an indenter with light for tooth monitoring (HAMILTOM) is proposed as an optical device. HAMILTOM could measure hardness of teeth in wet condition as a dark area while applying a load to dentins without drying. Therefore, HAMILTOM may realize hardness measurements of *in vivo* teeth in a clinical setting quantitatively.

**Aim:**

The aim of our study is to demonstrate the optical dentin hardness measuring device HAMILTOM using bovine dentin with different demineralization times and to evaluate the correlation between the dark areas measured by HAMILTOM and the Vickers hardness measured by the Vickers hardness tester.

**Approach:**

The samples were 20 bovine dentins. They were demineralized by a lactic acid solution with different times and divided into groups 1 and 2 of 10 samples each. In both groups, the dark areas and Vickers hardness were measured for each sample. Group 1 was used to obtain a calibration curve to calculate Vickers hardness from the dark area. Group 2 was used to validate the calibration curve obtained from the dentin samples of group 1.

**Results:**

The areas appearing black without a total internal reflection of the indenter measured by HAMILTOM increased as the demineralization time increased. Additionally, the Vickers hardness of group 2 calculated by the dark areas of group 2 and the calibration curve obtained in group 1 and the Vickers hardness of group 2 measured by the Vickers hardness tester were strongly correlated with a determination coefficient of 0.99.

**Conclusions:**

The results demonstrate that HAMILTOM may be a suitable alternative to the conventional method. Unlike the conventional method, which cannot be used for *in vivo* teeth, HAMILTOM holds potential to quantitatively evaluate the progress of caries in *in vivo* teeth.

## 1 Introduction

The world’s population is simultaneously increasing and aging. According to the WHO, one in six individuals will be 60 years or older by 2030, and the world’s population of people more than 60 years of age will reach 2.1 billion.^1^ Japan is a country with a remarkable aging population. Currently, more than one in four people are 65 years or older, and this is expected to increase to one in three by 2030.^2^ As life expectancy increases, people retain their teeth for longer.^3^

Root caries is a dental disease that affects more than one in three persons in the geriatric population.^4^ Root caries is commonly found on the exposed root surfaces or the margin of the cementoenamel junction.^5^ Root caries is defined as a cavitation below the cementoenamel junction not including the adjacent enamel. It is usually discolored, softened, ill-defined, and involves both cementum and underlying dentin.^6^ Enamel is stronger and more acid-resistant than any other dental tissue since it contains about 90% minerals.^7^ In comparison, cementum and dentin are composed of about 45% to 50% and 70% inorganic materials, respectively.^8^^,^^9^ In addition, the higher content of magnesium and carbonate makes cementum and dentin more soluble than enamel.^10^ Therefore, dentin on a root surface is not only more likely to develop and progress into caries than enamel but also more likely to cause tooth loss. The management of root caries is an important issue in the aging society because the risk of caries is higher as the number of untreated teeth increases.

Root caries is diagnosed by inspection and palpation. Indicators include the color, surface texture, and hardness of the lesion.^11^^,^^12^ Although the diagnosis is based on inspection and palpation, there is no clear color change in the initial root caries,^13^ and palpation is performed using a probe. Hence, the diagnosis is qualitative. Hardness testing is an indirect method to track changes in the mineral content of dentin.^14^^,^^15^ As a caries progresses, dentin becomes softer, but the diagnosis using a probe depends on the dentist’s sensitivity to pressure changes when inserting and removing the probe. In fact, the kappa statistics for the inspection and palpation for root caries are as low as 30% to 51%.^16^ Therefore, a method to objectively and quantitatively evaluate the activity and progress of root caries in a clinical setting is strongly desired.

A quantitative evaluation of the hardness of dentin may realize a more accurate assessment of the progression of root caries. Vickers or Knoop hardness has been customarily used as an indicator of tooth hardness.^17^ The Vickers hardness and Knoop hardness tests evaluate hardness by measuring the size of indentations after pressing an indenter of a quadrangular pyramid with a constant load on the target object. However, the sample must be dried prior to measurement because an indentation does not remain in samples with a large elastic deformation such as a carious tooth. Practically, a hardness measurement cannot be performed on an *in vivo* tooth as the tooth must be extracted prior to the evaluation. Cariotester is one technique to quantify the hardness of teeth in research, and the device has been registered as a medical device in Japan. In this technique, paint is applied to the tip of a metal indenter and the length of the part where the paint disappears after contacting the tooth is measured.^18^ This measurement procedure is complicated and requires a microscope for observations. Thus it is difficult to utilize in a clinical setting.

A technique and a device are proposed to easily measure the hardness of *in vivo* teeth using a light-emitting diode (LED). This technique is called “HAMILTOM,” which stands for “hardness meter using indenter with light for tooth monitoring.” HAMILTOM quantifies the hardness of dentin from the contact projection area (dark area) between the indenter and dentin when the indenter is pressed into the dentin. It is difficult to be used in proximal parts of teeth, but it could be used in root caries focused on in this study. Here the basic principle of HAMILTOM is demonstrated using bovine dentin samples with different demineralization times. The dark areas are measured by HAMILTOM, and the correlation with the Vickers hardness measured by a conventional Vickers hardness tester was evaluated.

## 2 Materials and Methods

### 2.1 Sample Preparation

The samples were 20 sound bovine dentins. Bovine dentins were used because they were easy to collect with a similar sample size. Since bovine dentins are larger than human dentins, it was easy to standardize the sample size and measure many points with one sample. These were used to evaluate the correlation between dark areas measured by HAMILTOM and Vickers hardness. Simulated models with various hardnesses were prepared by demineralizing sound dentin samples for different times to create caries. This study was performed after obtaining approval from the Animal Care and Use Committee, Osaka Dental University (Approval No. 19-12001).

**Fig. 1:**
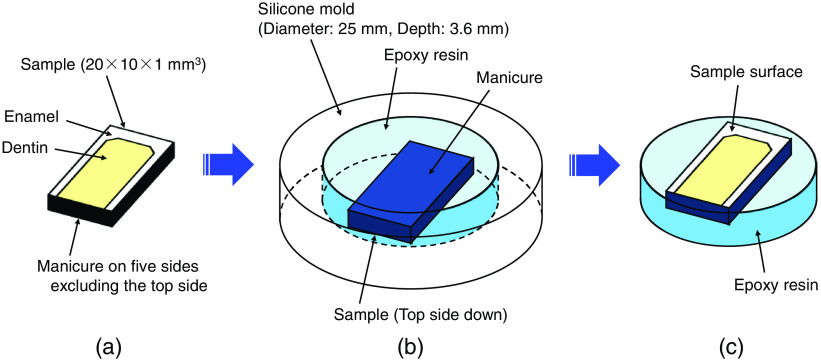
Procedures to prepare dentin samples: (a) cut extracted bovine tooth to approximately $20\times 10\times 1\;{\mathrm{mm}}^{3}$ samples. Apply manicure to the back and all sides of the dentin prior to embedding to protect the dentinal tubules from the epoxy resin solution. (b) Embed the sample in epoxy resin using a silicone mold and let cure for at least 24 h. (c) Remove the sample from the mold and mirror polish the surface under water injection using waterproof abrasive papers and a lapping film.

Figure 1 overviews the procedure to prepare dentin samples. Preparation includes cutting, the sample embedding in epoxy resin, curing for at least 24 h, and mirror polishing of the sample surface. First, the extracted bovine tooth was cut perpendicular to the running direction of the dentinal tubules. The cut dentin samples were $∼20\times 10\times 1\;{\mathrm{mm}}^{3}$. To protect the dentinal tubules from the epoxy resin solution, a manicure was applied to the back and all sides of the dentin samples prior to embedding. Second, the dentin samples were embedded in an epoxy resin (Crystal Resin, NISSIN RESIN, Japan) using silicone molds. The samples were cured for at least 24 h, and then they were removed from the molds. Third, the sample surfaces were mirror polished under water injection using waterproof abrasive papers #400, 800, and 2000 (Kohnan, Japan) and a lapping film #8000 (LF1D, Thorlabs, USA) to create a horizontal hardness measurement surface. The sample tilt was adjusted to 1 deg or less during polishing for the sample orientation of 90 deg to the indenter. In addition, ultrasonic cleaning was performed for 2 min using a desktop ultrasonic cleaner (B2210, Branson Ultrasonic, USA) to remove the resin pieces and the inorganic components of dentin generated by polishing from the dentinal tubules. The 20 dentin samples were divided into two groups of 10 samples each (groups 1 and 2).

The caries models were prepared by artificial demineralization. The dentin samples were immersed in a lactic acid solution.^19^^,^^20^ Lactic acid (20006-75, Nacalai Tesque, Japan) and distilled water were mixed in a beaker to prepare 1 L of 0.1 M demineralization solution. The beaker was placed in a constant temperature water bath (TM-3A, AS ONE, Japan) to hold the solution temperature at 37°C. The solution was stirred with a stirrer (HE-16GA, KPI, Japan) at the rotation speed of 1000 rpm to maintain a pH of 2. Next, each dentin sample was immersed in the solution for a predetermined time. The demineralization time was set to 0, 0.25, 0.5, 0.75, 1, 2, 4, 6, 12, or 24 h. After demineralization, the samples were washed with tap water and stored in saline (Otsuka Pharmaceutical, Japan) at 4°C.

A group contained 10 samples in which each sample was demineralized for different duration times. In groups 1 and 2, the dark areas and Vickers hardness were measured at three points for each sample. Group 1 was used to obtain a calibration curve to calculate Vickers hardness from the dark area. Group 2 was used to validate the calibration curve obtained from the dentin samples of group 1.

### 2.2 Optical Dentin Hardness Measuring Device

#### 2.2.1 Device setup and measurement principle

**Fig. 2:**
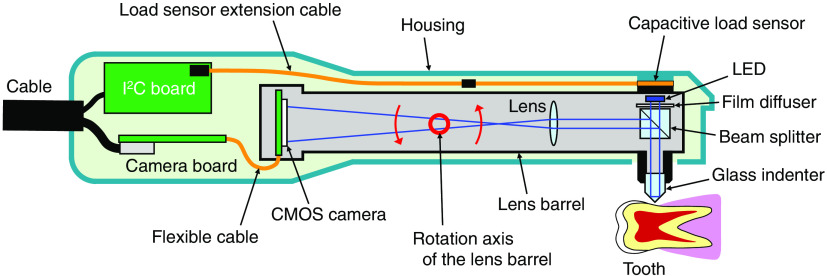
Schematic illustration of the HAMILTOM handpiece. Image of the tip of the glass indenter is projected onto the CMOS camera with a threefold magnification using light from the LED. By applying a load to the tip of the indenter, the lens barrel rotates around the rotation axis, and the lens barrel and the housing apply the load to the load sensor. The CMOS acquires an image of the indenter when the load reaches the set value.

Figure 2 shows a schematic illustration of HAMILTOM. HAMILTOM is a device that measures the hardness of dentin from the dark area, which is the contact area between the indenter and the dentin. HAMILTOM includes an optical system composed of an LED with a 455-nm center wavelength, a film diffuser (#17-682, Edmund Optics, USA), a beam splitter (#47-007, Edmund Optics), a transparent conical glass indenter with a 90-deg apex angle (#49-397, Edmund Optics), a lens with a 30-mm focal length (#45-134, Edmund Optics), and a CMOS camera (ID1MB-MDL-U, iDule, Japan) in a lens barrel. It should be noted that the purchased glass indenter originally had an aluminum mirror coating, and the coating was removed by immersing the indenter in hydrochloric acid before use. The LED light passing through the film diffuser was incident on the indenter. The reflected light was reflected toward the camera by the beam splitter, and the image of the tip of the glass indenter was projected on the CMOS camera using a lens with about threefold magnification. Out of the lens barrel, a capacitive load sensor with an 8-mm diameter and 0.3-mm thickness [SingleTact S8-1N, Pressure Profile Systems (PPS), USA], a load sensor extension cable (SingleTact Tail-Extender, PPS), and an $I^{2}C$ digital interface board (SingleTact Standard Electronics, PPS) were placed in a handpiece housing. By applying a load to the tip of the indenter, the lens barrel rotates around the rotation axis. Then the lens barrel and the housing applied the load to the load sensor. The load applied to the sensor was continuously monitored and recorded on a tablet PC (Surface Go 2, Microsoft, USA) through the $I^{2}C$ digital interface board and a microcontroller (PSoC 5LP, Cypress Semiconductor, USA). An image of the indenter when the load reached the set value was acquired automatically by the CMOS camera using in-house software.

**Fig. 3:**
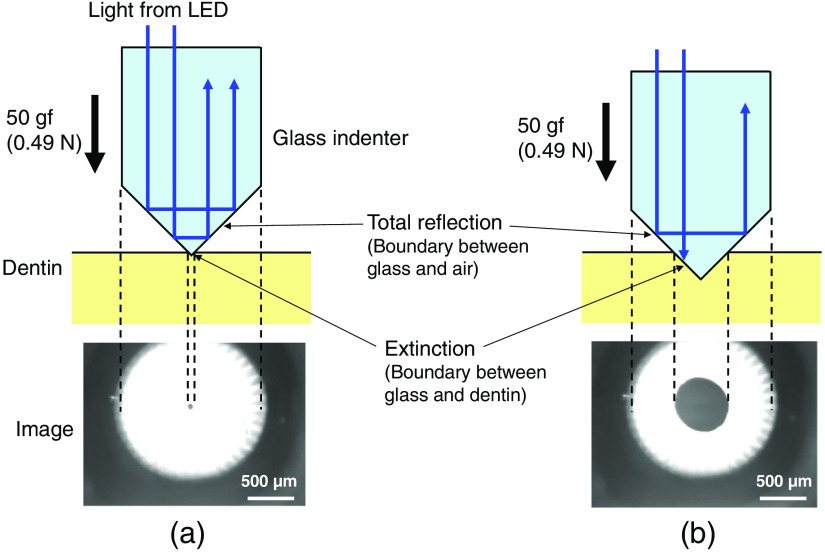
Principle of measuring the dark area between the glass indenter and dentin. When the indenter comes into contact with dentin, a total internal reflection of LED light does not occur, and the indenter at the contact area appears dark because the refractive indices of the glass indenter and dentin are similar. (a) Dark area for sound dentin, which is hard, is small at a constant load of 50 gf (∼0.49  N). (b) The dark area for demineralized dentin is larger for a given load because the demineralized dentin is softer than sound dentin.

Figure 3 shows the measurement principle of HAMILTOM. When the indenter is not in contact with the dentin, the indenter appears bright because a total internal reflection occurs at the boundary between the glass indenter and the air. In contrast, when the indenter comes into contact with the dentin, a total internal reflection does not occur. Thus the indenter at the contact area appears dark because the refractive index of N-BK7 (1.52), which is the material of the glass indenter,^21^ and that of dentin (1.54) are close.^22^ For a constant contact load, the dark area should be larger when the dentin is softer. Therefore, the hardness of the dentin can be assessed by measuring the dark area when a constant load is applied to the tip of the indenter. Dentin becomes softer as caries progresses. Hence the dark area should indicate the degree of caries progression because the dark area should increase compared to sound dentin. It is necessary to emphasize that the dark areas are not the size of the indentation and the influence of the water exuded by the contact of the indenter is significant, and it is considered that the indentation does not remain due to the elastic recovery of the dentin after removing the indenter.

#### 2.2.2 Calculation of the dark area

**Fig. 4:**
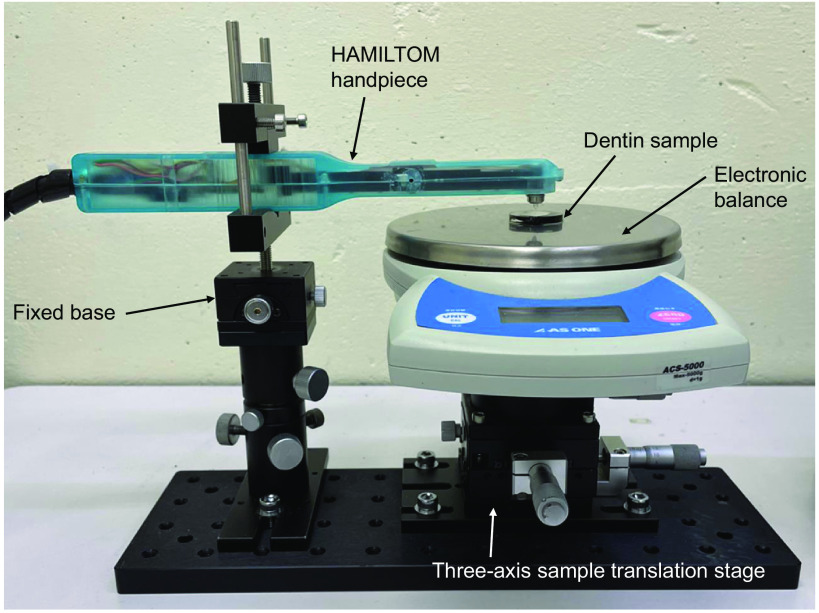
Image of the experimental system for the measurement of dark areas by HAMILTOM. HAMILTOM is fixed at the base, and the dentin sample is located directly below the indenter. The stage is slowly raised vertically, and an image acquired with the CMOS camera when the load reaches 50 gf (∼0.49  N). Load is also measured by an electronic balance to confirm the accuracy of the load measured by the load sensor.

The dark areas were measured using HAMILTOM for the dentin sample at each demineralization time. Prior to each measurement, the dentin sample was immersed in saline for at least 24 h to make it moist. Figure 4 shows a photograph of the experimental setup to measure the dark areas using HAMILTOM under a stable condition. HAMILTOM was fixed so that the tip of the indenter was oriented vertically downward. The excess surface moisture was blown off with air, and the dentin sample was placed on the stage so that it was located directly below the indenter. The threshold of the load sensor when calculating the dark area was set to 50 gf. The stage was manually raised in the vertical direction. The CMOS camera acquired an image once the load reached the specified value. The load was also measured by an electronic balance (ACS-5000, AS ONE, Japan) to confirm the accuracy of the load measured by the load sensor. The accuracy of the electronic balance was confirmed before and after the experiments using a standard weight set (3-9951-04, AS ONE). Before the experiment, a coefficient to be multiplied to the measured value of the load sensor was adjusted to match the load measured with the electronic balance. After setting the dentin sample on the electronic balance, the electronic balance was reset to zero each time because the weight of each sample was slightly different. Images of the dark area were obtained at three different positions for each dentin sample. After each measurement, a lens cleaning paper (EK1546027S, Tiffen, USA) with ethanol was used to remove the water and deposits on the surface of the glass indenter.

To calculate the dark area, a reference image was obtained before the indenter was brought into contact with the sample. When each reference image was acquired, the load measured with the load sensor was reset to zero to cancel the long-term drift of the sensor. Next, an image was acquired after the indenter was brought into contact with the sample. Then the reference was subtracted. A binarized image was created using a threshold of 50% of the maximum brightness of the subtracted image. In the binarized image, the contact area appears as bright pixels. Finally, the dark area was calculated from the number of the bright pixels in the binarized image.

### 2.3 Vickers Hardness

The Vickers hardness was measured using a Vickers hardness tester (HMV-G30S, Shimadzu, Japan) for each sample, which had different demineralization times. Conventionally, the Vickers hardness is measured in dry conditions because indentations do not clearly remain on a sample with a large elasticity such as demineralized dentin. The dentin samples in this study were dried in a 23°C indoor environment at 25% humidity for 18 h before the hardness measurements. The test load was set to 500 gf (∼4.9  N). The load holding time was 10 s, and indentations were made on the dentin samples. The Vickers hardness was calculated by measuring the length of the diagonal line of the indentation. The indentation was observed with the microscope of the hardness tester. The Vickers hardness of each sample was measured at three different positions. The measurement points were within a 5-mm diameter with respect to the measurement points with HAMILTOM.

### 2.4 Evaluation

First, the dark area and the Vickers hardness of the 10 dentin samples in group 1 were measured with HAMILTOM and the Vickers hardness tester, respectively. Next, a calibration curve was created to calculate the Vickers hardness from the dark area measured with HAMILTOM. Then the dark area and the Vickers hardness of the ten samples in group 2 were measured with HAMILTOM and the hardness tester, respectively. Using the aforementioned calibration curve, the Vickers hardness was measured using both HAMILTOM and the Vickers hardness tester. The correlation between both methods was evaluated.

## 3 Results

**Fig. 5:**
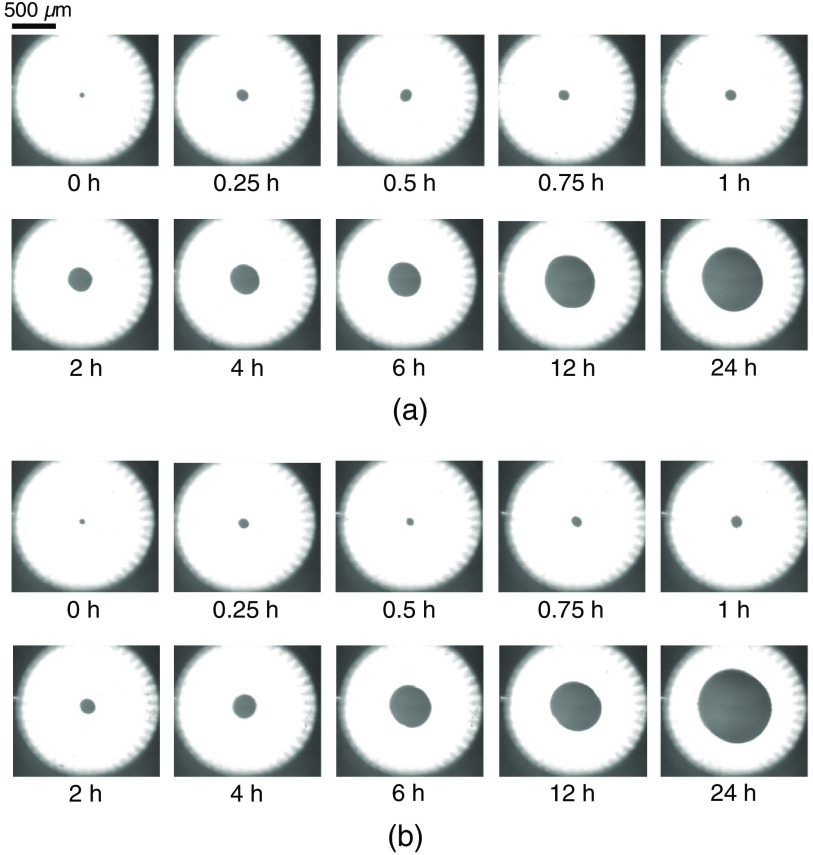
Typical images of reflected light from the glass indenter taken with the CMOS camera. Images for dentin samples with different demineralization times (0, 0.25, 0.5, 0.75, 1, 2, 4, 6, 12, or 24 h) are shown for (a) group 1 and (b) group 2, respectively.

Figure 5 shows typical images of the reflected light from the indenter when measuring the dark areas of the samples in groups 1 and 2. When the dark areas were measured, the load values measured by the electronic balance were 46 to 54 gf. The surface quality of the glass indenter did not change in the repeated measurement in this study because the threshold of the load sensor was set to 50 gf for reducing the burden on the indenter. The areas appearing black without a total internal reflection of the indenter increased as the demineralization time increased for both groups. In the images of the reflected light from the indenter, any dentinal tubules were not observed. It is considered that there was no influence of the porosity of the dentin to the dark area.

**Fig. 6:**
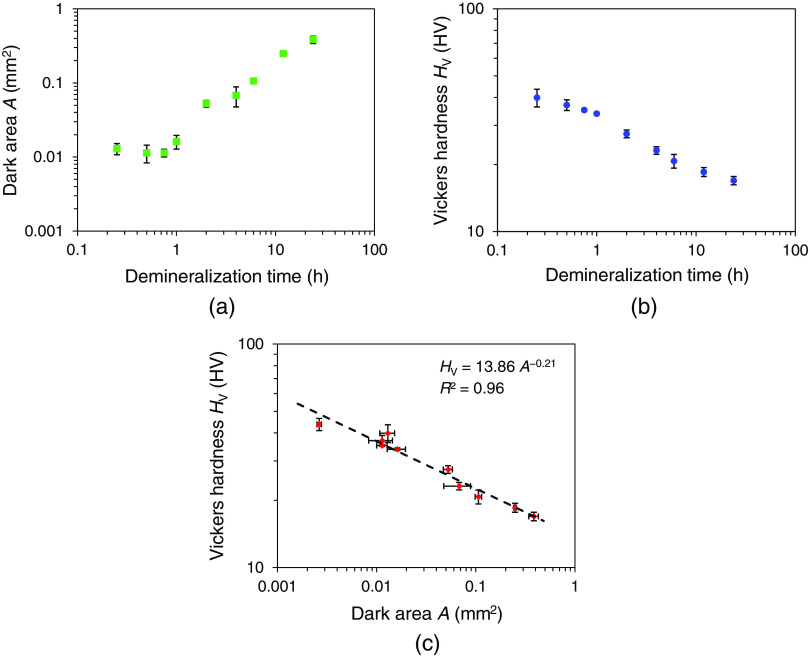
Dark area, Vickers hardness, and their relationship for the samples in group 1. Each graph shows the mean and standard deviation of three measurements. (a) Changes in the dark area with different demineralization times. (b) Changes in the Vickers hardness with different demineralization times. (c) Relationship between the dark area and the Vickers hardness. Dashed line shows the approximate expression. A determination coefficient is also shown.

Table 1 shows the changes in the dark area and the Vickers hardness for group 1 with different demineralization times, and Fig. 6 plots the relationship between the dark area A and the Vickers hardness $H_{V}$. As the demineralization time increased, the dark areas increased. Additionally, the Vickers hardness decreased to less than half of that of the sound dentin at a demineralization time of 6 h. Evaluating the correlation between the dark areas and the Vickers hardness using a power approximation gave a determination coefficient of 0.96.

**Table 1:** Dark area and the Vickers hardness of the samples in group 1. Mean and standard deviation (SD) of three measurements are shown.

		Demineralization time (h)									
		0	0.25	0.5	0.75	1	2	4	6	12	24
Dark area (${\mathrm{mm}}^{2}$)	Mean	0.0026	0.013	0.011	0.011	0.016	0.053	0.07	0.11	0.25	0.39
	SD	0.0001	0.002	0.003	0.001	0003	0.006	0.02	0.01	0.01	0.04
Vickers hardness (HV)	Mean	44	40	37	35	33	27	23	21	18	17
	SD	3	4	2	1	1	1	1	1	1	1

**Fig. 7:**
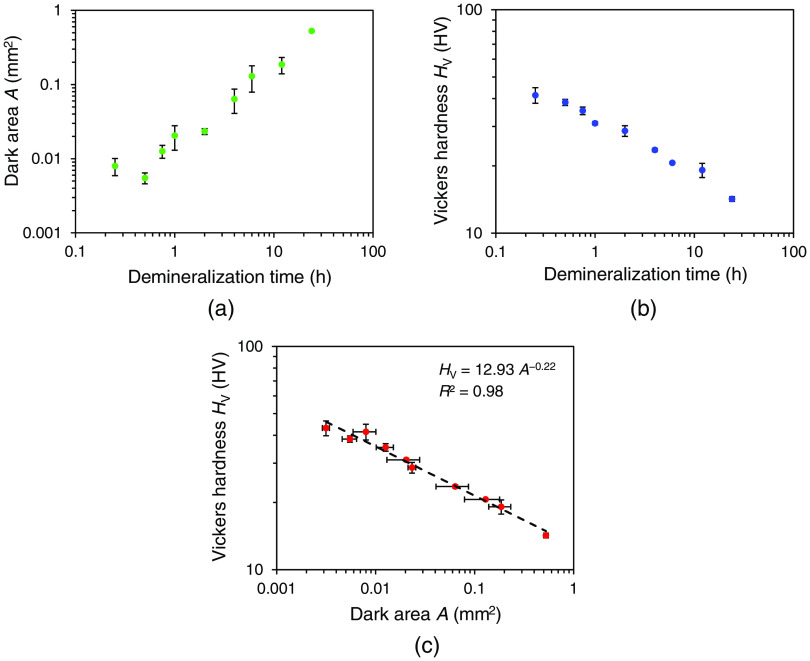
Dark area, Vickers hardness, and their relationship for the samples in group 2. Each graph shows the mean and standard deviation of three measurements. (a) Changes in the dark area with different demineralization times. (b) Changes in the Vickers hardness with different demineralization times. (c) Relationship between the dark area and the Vickers hardness. Dashed line shows the approximate expression. A determination coefficient is also shown.

Table 2 and Fig. 7 show similar data for group 2. Both groups display the same trend. The determination coefficient for group 2 was 0.98.

**Table 2:** Dark area and the Vickers hardness of the samples in group 2. Mean and SD for three measurements are shown.

		Demineralization time (h)									
		0	0.25	0.5	0.75	1	2	4	6	12	24
Dark area (${\mathrm{mm}}^{2}$)	Mean	0.0032	0.008	0.006	0.011	0.013	0.023	0.06	0.13	0.19	0.53
	SD	0.0002	0.002	0.003	0.009	0.003	0.002	0.02	0.05	0.05	0.01
Vickers hardness (HV)	Mean	43	41	38	35	31	29	24	21	19	14
	SD	3	3	2	1	1	2	1	1	1	1

**Fig. 8:**
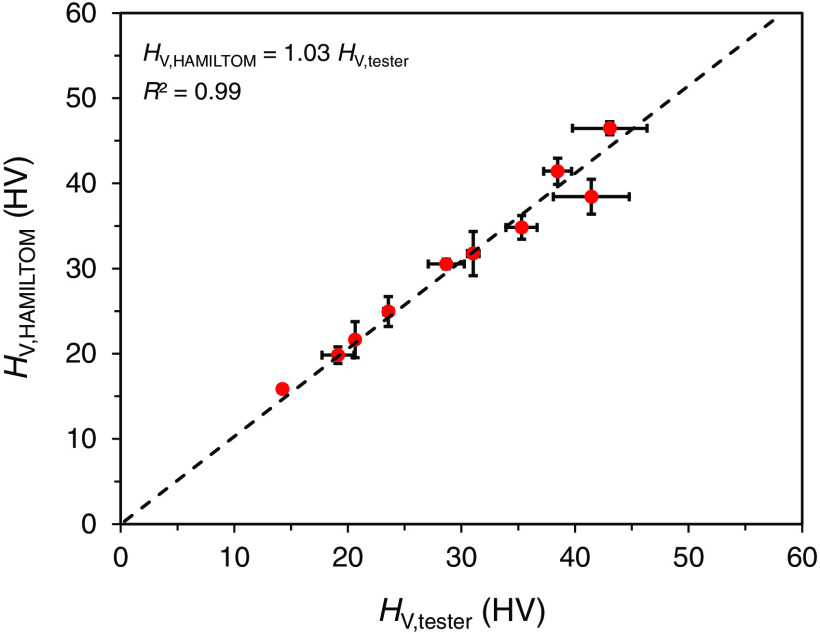
Relationship between the Vickers hardness $H_{V,\mathrm{tester}}$ of the samples in group 2 measured with the Vickers hardness tester and the Vickers hardness $H_{V,\mathrm{HAMILTOM}}$ calculated by the dark area of the samples in group 2 using the calibration curve obtained from the samples in group 1. The mean and standard deviation of three measurements, approximate expression (dashed line), and determination coefficient are shown.

Figure 8 plots the relationship between the Vickers hardness of group 2 calculated by the dark areas of group 2 and the calibration curve obtained in group 1 and the Vickers hardness of group 2 measured by the Vickers hardness tester. There is a strong correlation with a determination coefficient of 0.99. Furthermore, p-value of Pearson’s correlation coefficient was $3\times 10^{-7}$, and the p-value was smaller than the significance level of 5%. Therefore, it was suggested that the correlation shown in Fig. 8 was significant and the Vickers hardness can be calculated by measuring the dark areas using HAMILTOM.

## 4 Discussion

In this study, the dark areas and the Vickers hardness were measured for bovine dentins with different demineralization times, and the correlation coefficient was evaluated. HAMILTOM measurement in group 2 gave direct Vickers hardness based on the calibration curve obtained in group 1, and the correlation between both methods showed a strong determination coefficient of 0.99. Theoretically formulating the correlation between dark areas and the Vickers hardness may make it possible to estimate the Vickers hardness from the dark area measured using HAMILTOM. Groups 1 and 2 have similar demineralized conditions since they were demineralized by the same procedure. The actual caries process includes many factors, such as microbiology, saliva, tooth mineral composition, tooth ultrastructure, diffusion processes, kinetics of demineralization, and the reversal of demineralization, which is known as remineralization and factors that contribute to the reversal process.^23^ The actual demineralization reaction is not driven by lactic acid, which was employed in this experiment, but is controlled mainly by bacteria such as *Streptococcus mutans* and *Lactobacillus* fermenting food and producing acid to dissolve tooth minerals.^23^ It has also been reported that the origins of crown caries and root caries differ. Plaque accumulation, frequency of sugar intake, decreased saliva volume, fluoride exposure, and low standard of living are factors for both crown caries and root caries, whereas gingival recession, attachment loss, and aging also contribute to root caries.^24^ Therefore, the correlation and the validity of the power approximation formula must be further evaluated to apply the power approximation formula derived in this study to actual caries of human dentins. An experimental system that reproduces the actual caries process such as by a pH cycle test is planned to make the demineralization process more similar to the actual. Evaluation with not only the index of demineralization time but also the relationship with the amount of minerals is needed, and an experiment to evaluate the degree of calcification by X-ray micro-computed tomography (micro-CT) is also planned.

This study confirmed the correlation between the dark areas measured by HAMILTOM and the Vickers hardness measured by the Vickers hardness tester (Figs. 6 and 7). The change in the dark areas increased significantly when the demineralization time was 2 h or more compared to the change in the Vickers hardness when the demineralization time was <2h for both groups 1 and 2 (Tables 1 and 2). This may be related to the measurement conditions of the dentin samples. Regarding the dark areas, the excess surface moisture was removed by blow drying with air after removing the dentin sample from saline, and the measurement was performed in a wet condition. In contrast, the Vickers hardness was measured after the dentin sample was dried in an indoor environment for 18 h because an indentation did not remain due to the elastic recovery after the indenter was pulled out in the wet condition. Dentin is composed of inorganic components, collagen fibers, and water. The volume of the eluted inorganic components is replaced by water in the demineralization reaction.^25^ Furthermore, the demineralized dentin is dehydrated. The unbound water between the collagen molecules is lost, and the collagen fibers are aggregated by peptide bonds. A longer demineralization time increased the darker areas due to the increased water volume in the dentin. However, the change in the water content was offset by drying the dentin sample in the Vickers hardness, and the demineralized layer was condensed and showed a smaller change than the dark areas. Another reason for difference of increase of the dark areas depends on the demineralization time. Figures 6 and 7 show that HAMILTOM might not be very sensitive within a demineralization time of 1 h, and improvement is necessary. Due to problems with the design of the optical system, it has been confirmed that a black spot appears at the tip of the indenter before the indenter comes into contact with samples. The size of the black spot in this experiment was $2\times 10^{-3}\;{\mathrm{mm}}^{2}$, and the dark areas cannot be calculated accurately when the area of the black spot exceeds the dark areas on dentin samples. Since the dark area was small in the sample with a short demineralization time, it could be easily affected by the black spot. In order to make the area of black spot smaller than the dark area of sound dentin or initial caries, improvement has been tried by designing the optical system and indenter. A method of suppressing the influence of black spots by increasing the threshold of the load sensor is also conceivable, but since there is a trade-off relationship between invasiveness and measurement accuracy, invasiveness might increase. It is necessary to discuss whether the current invasiveness is acceptable, but it is needed to consider whether it is possible to measure the dark area with a lower load than 50 gf by making the black spot smaller as a less invasive measurement condition.

HAMILTOM should realize practical use as a method to objectively and quantitatively evaluate the activity and progress of caries, especially root caries. Hence, the diagnostic device may support a treatment plan. In particular, it may be able to determine whether the detected caries should be removed or not. The conventional Vickers hardness values of sound dentin have been reported as 50 to 60.^26^ The hardness of dentin changes continuously as caries progresses. In the process, it has been reported that the area where caries dentin should be removed or preserved can be divided by a Vickers hardness of 30 to 40. Consequently, the Vickers hardness may be used as an index for caries removal.^27^ Regions where the Vickers hardness is 30 or more show small changes in the dark area. The relationship between the dark area and the Vickers hardness deviates from an inverse proportional relationship in some points. To use HAMILTOM as a diagnostic device of dentin hardness and the converted Vickers hardness to determine the necessity for caries, the measurement accuracy of HAMILTOM must be improved. In this study, the average maximum error of Vickers hardness measured by the Vickers hardness tester was 5%, whereas the average maximum error of converted Vickers hardness measured by HAMILTOM was 8%. An error caused by the sample is considered as a common error factor, and it is possible that the hardness variation in the sample had an effect. Furthermore, as an error in HAMILTOM, a load error from 46 to 54 gf could be considered and improvement of the precision of the load is needed.

The difference of situations between *in vitro* and *in vivo* should be discussed. In terms of the sample orientation, in this study, the sample orientation was 90 deg to the indenter by adjusting the sample tilt and device orientation. However, the angle could change* in vivo* situation because the angle might depend on dentists. In this study, it has been confirmed that if the angle of the indenter is about 5 deg to the sample surface, the effect on the measurement accuracy is negligible, but if the angle is larger than that, the dark area is not a precise circle and expands. Therefore, a method of estimating the angle of the indenter from the circular shape and the dark area without the tilt of the indenter has been considered. In addition to the sample orientation, the surface condition of dentin and the degree and size of demineralization could be different between *in vitro* and *in vivo*. In actual clinical practice, it would be inevitable that the obtained values by HAMILTOM differ depending on the condition and size of caries, but even if this is taken into consideration, the current uncertainty of caries diagnosis could be improved, it might be useful in determining the need for intervention. The purpose of HAMILTOM is to reduce the uncertainty of inspection with an operator by quantifying the degree of caries as hardness. The quantification could improve the uncertainty and might be used for definite diagnosis in cases where dentists have difficulty making decisions.

The evaluation of dentin hardness in wet conditions using HAMILTOM suggests that the problem of the conventional hardness measuring device can be solved. It could be possible to measure dark areas in dry conditions as Vickers hardness measurement, but the advantage of HAMILTOM is that dark areas can be measured in wet conditions without removing dentins, which is superior to the Vickers hardness measurement. This is because the Vickers hardness is measured by applying a constant load and measuring the indentation after removing the indenter, whereas HAMILTOM measures the dark area while applying a load to dentins without drying. Assuming actual practical use of HAMILTOM, the relationship between the conditions of loading time and speed and the measurement accuracy will be evaluated in the future. If the hardness of root caries can be easily and quantitatively evaluated, the activity and progression of root caries can be assessed more accurately. The Vickers hardness is a conventional method to determine tooth hardness, but the tooth must be removed before the measurement. Hence, it is not applicable to *in vivo* teeth. In contrast, HAMILTOM developed in this study can measure the dark areas in wet dentin samples. Since HAMILTOM is supposed to be used in a handpiece type clinically, the easy hardness measurement is also expected. A study of comparison for diagnostic accuracy between HAMILTOM and classical methods, such as inspection and palpation, for root caries could be planned after HAMILTOM becomes available for clinical use in the future. HAMILTOM may realize hardness measurements of *in vivo* teeth in a clinical setting and quantitatively evaluate the occurrence and progression of dental caries by measuring the temporal change of the hardness.

## 5 Conclusions

This study evaluated the dark areas of bovine dentins with various demineralization times. HAMILTOM easily measured the dentin hardness. Next, the correlation between the dark area measured by HAMILTOM and the Vickers hardness measured by the Vickers hardness tester was evaluated. As the demineralization time increases, the areas appearing black without a total internal reflection of the indenter increase. Additionally, the Vickers hardness of group 2 calculated by the dark areas of group 2 and the calibration curve obtained in group 1 and the Vickers hardness of group 2 measured by the Vickers hardness tester are strongly correlated with a determination coefficient of 0.99. The results demonstrate that HAMILTOM may be a suitable alternative to the conventional method. Unlike the conventional method, which cannot be used for *in vivo* teeth, HAMILTOM holds potential to quantitatively evaluate the progress of caries in *in vivo* teeth. In the future, HAMILTOM may become a new hardness diagnostic method for root caries.

## Code, Data, and Materials Availability

Data underlying the results presented in this paper are not publicly available at this time but may be obtained from the authors upon reasonable request.
